# Managing argon interference during measurements of ^18^O/^16^O ratios in O_2_ by continuous-flow isotope ratio mass spectrometry

**DOI:** 10.1007/s00216-022-04184-3

**Published:** 2022-07-16

**Authors:** Charlotte E. Bopp, Jakov Bolotin, Sarah G. Pati, Thomas B. Hofstetter

**Affiliations:** 1 Eawag, Swiss Federal Institute of Aquatic Science and Technology, CH-8600 Dübendorf, Switzerland; 2 Institute of Biogeochemistry and Pollutant Dynamics (IBP), ETH Zürich, CH-8092 Zürich, Switzerland; 3 Department of Environmental Sciences, University of Basel, CH-4056 Basel, Switzerland

**Keywords:** Isotope ratio mass spectrometry, Oxygen isotope fractionation, Kinetic isotope effects, Dissolved oxygen

## Abstract

**Abstract:**

Monitoring changes in stable oxygen isotope ratios in molecular oxygen allows for studying many fundamental processes in bio(geo)chemistry and environmental sciences. While the measurement of $^{18}$O/$^{16}$O ratios of $\mathrm {O}_{2}$ in gaseous samples can be carried out conveniently and from extracting moderately small aqueous samples for analyses by continuous-flow isotope ratio mass spectrometry (CF-IRMS), oxygen isotope signatures, $\updelta ^{18}$O, could be overestimated by more than 6$\permille$ because of interferences from argon in air. Here, we systematically evaluated the extent of such Ar interferences on $^{18}$O/$^{16}$O ratios of $\mathrm {O}_{2}$ for measurements by gas chromatography/IRMS and GasBench/IRMS and propose simple instrumental modifications for improved Ar and $\mathrm {O}_{2}$ separation as well as post-measurement correction procedures for obtaining accurate $\updelta ^{18}$O. We subsequently evaluated the consequences of Ar interferences for the quantification of O isotope fractionation in terms of isotope enrichment factors, $\upepsilon _{\mathrm {O}}$, and $^{18}$O kinetic isotope effects ($^{18}$O KIEs) in samples where $\mathrm {O}_{2}$ is consumed and Ar:$\mathrm {O}_{2}$ ratios increase steadily and substantially over the course of a reaction. We show that the extent of O isotope fractionation is overestimated only slightly and that this effect is typically smaller than uncertainties originating from the precision of $\updelta ^{18}$O measurements and experimental variability. Ar interferences can become more relevant and bias $\upepsilon _{\mathrm {O}}$ values by more than $2\permille$ in aqueous samples where fractional $\mathrm {O}_{2}$ conversion exceeds 90%. Practically, however, such samples would typically contain less than 25 $\upmu$M of $\mathrm {O}_{2}$ at ambient temperature, an amount that is close to the method detection limit of $^{18}$O/$^{16}$O ratio measurement by CF-IRMS.

**Graphical abstract:**

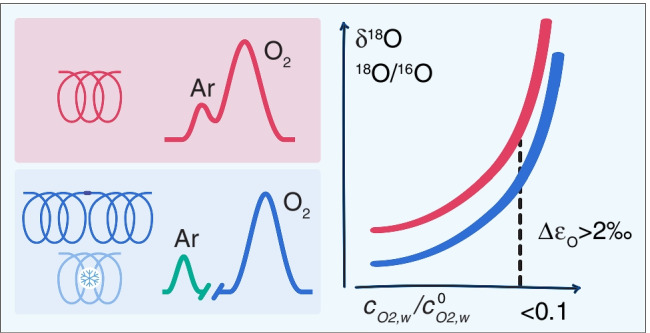

**Supplementary Information:**

The online version contains supplementary material available at 10.1007/s00216-022-04184-3.

## Introduction

Changes in $^{18}$O/$^{16}$O and $^{17}$O/$^{16}$O ratios of dioxygen are used to study fundamental processes involving the formation and consumption of molecular $\mathrm {O}_{2}$ in (bio)geochemistry, biochemistry, and environmental science. On an ecosystem or even planetary scale, oxygen isotope fractionation of $\mathrm {O}_{2}$ between reservoirs are dominated by photosynthesis and respiration enabling the estimation of primary productivity [1–6]. On a molecular scale, kinetic and equilibrium isotope effects of enzymatic and chemical reactions reveal the mechanisms and kinetics of the electron and proton transfer steps associated with O–O bond cleavages [7–15].

Typically, the stable isotope ratios of $\mathrm {O}_{2}$ are determined by isotope ratio mass spectrometry (IRMS) directly from gaseous samples or in the headspace of dissolved $\mathrm {O}_{2}$ samples after removal of other atmospheric gases. In applications of traditional, dual inlet IRMS (DI/IRMS), this sample cleanup requires extensive offline sample preparation before injection [16–19]. Continuous-flow methods, by contrast, require specialized autosampler devices and instrumental modifications not available in many stable isotope laboratories [20, 21] and are often limited to large sample volumes of up to 250 mL [22, 23]. Recently, we proposed a method for sensitive and robust quantification of oxygen isotope ratios by gas chromatography coupled to isotope ratio mass spectrometry (GC/IRMS), [13, 24] a popular and widely available instrumental setup for continuous-flow isotope ratio mass spectrometry (CF-IRMS). Moreover, this setup allows working with smaller sample volumes of 11 mL which is more suitable for the laboratory-scale experiments in which reactant materials such as enzymes are limited. However, this and other CF-IRMS approaches [20, 22–25] so far did not systematically address issues from the co-elution of Ar with $\mathrm {O}_{2}$ even though Ar has been shown to interfere with the accurate determination of $\updelta ^{18}$O in DI/IRMS [26–29].

Argon is difficult to separate from $\mathrm {O}_{2}$, both chromatographically and cryogenically [30, 31]. Previous works with DI/IRMS reported positive correlations of measured $\updelta ^{18}$O and $\updelta ^{17}$O values with the ratio of partial pressure of Ar relative to $\mathrm {O}_{2}$ (i.e. Ar:$\mathrm {O}_{2}$ ratios) and illustrate Ar interferences of very different magnitude (Table S1) [26–29]. The origin of Ar interferences is not fully understood but it is attributed to a reduction of ionization efficiencies of the different $\mathrm {O}_{2}$ isotopologues in the presence of Ar [1, 27]. Even at low atmospheric Ar concentrations of 0.934 vol-%, [32] Ar interferes with precise quantification of $\mathrm {O}_{2}$ isotope ratios. Procedures for eliminating Ar interferences with DI/IRMS are known, such as corrections on the basis of linear correlation between deviations of $\updelta ^{18}$O or $\updelta ^{17}$O and Ar:$\mathrm {O}_{2}$ ratios [1, 26–29]. By contrast, there is no comprehensive and equally systematic assessment of the extent of Ar interferences of O isotope ratio measurements by CF-IRMS. It is therefore unknown whether DI/IRMS-based procedures for error correction apply equally well to CF-IRMS instrumentation and whether Ar interferences can be eliminated effectively through gas chromatography. In fact, volumetric Ar:$\mathrm {O}_{2}$ ratios can vary considerably, especially in environments and experiments, where $\mathrm {O}_{2}$ is consumed while Ar contents remain unchanged. Quantitative interpretation of O isotope fractionation and the corresponding $^{18}$O kinetic isotope effects ($^{18}$O KIEs) under such circumstances, for example, during chemical and enzymatic activation of O_2_ [8–14] could therefore be compromised by Ar interferences.

The goals of this study were (i) to show how to prevent Ar interference in continuous-flow $^{18}$O/$^{16}$O ratio mass spectrometry of $\mathrm {O}_{2}$ by simple and ready-to-establish adjustments in instrument setup as well as (ii) to illustrate how to correct for Ar interference in the evaluation of $^{18}$O/$^{16}$O data. To that end, we determined the extent of Ar interference on $^{18}$O/$^{16}$O measurements of $\mathrm {O}_{2}$ in gaseous samples with different Ar:$\mathrm {O}_{2}$ ratios for two popular and widely used instrumental configurations for CF-IRMS, namely GC/IRMS and GasBench/IRMS. On the one hand, we minimized Ar interferences by improving the chromatographic separation of Ar and $\mathrm {O}_{2}$ through increased column length on GC/IRMS devices and with cooled GC columns of GasBench/IRMS systems. On the other hand, we explored the utility of post-measurement corrections of $\updelta ^{18}$O values by manual peak integration and linear correction factors that account for Ar:$\mathrm {O}_{2}$ ratios. Finally, we evaluated the relevance of Ar interferences on $^{18}$O/$^{16}$O measurements for quantifying the extent of O isotope fractionation in $\mathrm {O}_{2}$ both from a theoretical perspective and with experiments on the enzymatic reduction of $\mathrm {O}_{2}$.

## Experimental section

A complete list of chemicals is provided in Section S1 in the Supplementary Information.

### Preparation of gaseous samples with different Ar: O_2 _ratios

For analysis of $^{18}$O/$^{16}$O ratios of $\mathrm {O}_{2}$ by GC/IRMS, 12 mL crimp vials were exposed to $\mathrm {N}_{2}$ inside an anaerobic glove box with $\mathrm {N}_{2}$ atmosphere (99.999%) (MBraun, residual $\mathrm {O}_{2}$ content $<1$ ppm) and sealed with butyl rubber septa. Once removed from the glovebox, 0.5, 1, or 1.5 mL of synthetic air (20 vol-% $\mathrm {O}_{2}$) or ambient air were injected into the crimp vial with a gas-tight syringe followed by the addition of 20 to 800 $\upmu$L Ar gas (99.999%) leading to volumetric Ar:$\mathrm {O}_{2}$ ratios between 0 and 0.8 at different total $\mathrm {O}_{2}$ concentrations in the sample vials (0.3, 1.5, or 3.4 mM $\mathrm {O}_{2}$). Samples were thereafter filled to a constant pressure of 2 bar with $\mathrm {N}_{2}$ gas. Blanks consisted of identical crimp vials filled with 2 bars of $\mathrm {N}_{2}$. For $^{18}$O/$^{16}$O ratio measurements on a GasBench/IRMS, 12 mL Exetainer vials (Labco Limited) with screw caps and butyl rubber septa were purged with He gas with a double-needle setup for 1 h. Purged Exetainers were considered blank samples. After purging, 25–250 $\upmu$L synthetic or ambient air and 0–25 $\upmu$L Ar gas were injected to the Exetainer with a gas-tight syringe through the butyl rubber septum. Exetainers contained Ar:$\mathrm {O}_{2}$ ratios of 0 to 0.8 and $\mathrm {O}_{2}$ concentrations of 18 to 178 $\upmu$M.

### Oxygen isotope fractionation experiments

The enzymatic reduction of $\mathrm {O}_{2}$ by glucose oxidase was carried out in two types of 50 mM sodium acetate solutions buffered at pH 5.0 following procedures described in Pati et al. [24] and summarized below. The first type of buffer solution was equilibrated with ambient air at room temperature. The second type, referred to as Ar-free buffer, was purged with synthetic air after heating in a serum bottle to remove dissolved Ar. Ar-free buffer was quickly transferred from the serum bottle to the sample vials under ambient atmosphere. 12 mL crimp vials were completely filled with the respective buffer and closed without headspace. A 50 mM glucose stock solution was prepared in sodium acetate buffer and equilibrated for 7 days to ensure equilibrium between $\upalpha$- and $\upbeta$-D-glucose.

Assays were prepared by adding 10–67 $\upmu$L of the equilibrated glucose solution to sodium acetate buffer solutions. Thereafter, the 12 mL crimp vials were closed without headspace, resulting in initial glucose concentrations between 40 and 280 $\upmu$M. The $\mathrm {O}_{2}$ reduction reaction was initiated by adding 24 $\upmu$L of a 6 mg mL$^{-1}$ glucose oxidase stock solution through the butyl rubber septum (12 $\upmu$g mL$^{-1}$ final concentration). Controls were prepared identically except for the addition of enzyme.

Dissolved $\mathrm {O}_{2}$ concentrations were monitored continuously in stirred vials with a needle-type oxygen microsensor (PreSens - Precision Sensing GmbH) immersed into the solution. After $\mathrm {O}_{2}$ consumption stopped, a $\mathrm {N}_{2}$ headspace was introduced by manually replacing 3 mL of aqueous solution with 3 mL of $\mathrm {N}_{2}$ under a constant pressure of 2 bar. Partitioning of dissolved $\mathrm {O}_{2}$ was facilitated by 30 min horizontal shaking at 200 rpm with the vials kept upside down [13, 24]. All samples from the enzymatic $\mathrm {O}_{2}$ reduction experiment were analysed for $^{18}$O/$^{16}$O ratios with a GC/IRMS.

### Instrumental analysis

Gaseous and headspace samples in crimp vials were analysed by GC/IRMS consisting of a gas chromatograph coupled via a Conflo IV interface to a Delta V Plus isotope ratio mass spectrometer (Thermo Fisher Scientific). Our previously introduced instrumental procedure [24] was modified to enable repeated injections from the same headspace sample as documented in Section S2. All samples were injected on-column by a PAL RSI (CTC Analytics) with a 2.5 mL gas-tight headspace syringe (Gauge 23, CTC Analytics). Prior to sample loading, the syringe was flushed with $\mathrm {N}_{2}$ gas for 1 min. For each measurement, 500 $\upmu$L of gaseous or headspace sample was injected into the split injector with Helium (99.999%) as the carrier gas and a split flow of 40 mL min$^{-1}$. Chromatographic gas separation was carried out with one and two 30 m Rt-Molsieve 5 Å  PLOT columns (Restek from BGB Analytik; 30 m x 0.32 mm ID, 30 $\upmu$m film thickness) and a PLOT column particle trap (Restek from BGB Analytik; 2.5 m x 0.32 mm ID). The columns were kept at a constant temperature of 30 ${}^{\circ }$C and an inlet pressure of 80 and 115 kPa for the 30 m and 60 m column setup, respectively.

Gaseous samples in Exetainers were placed on the autosampler of a GasBench II system coupled via a Conflo IV interface to a Delta V Plus isotope ratio mass spectrometer (Thermo Fisher Scientific). Sample gas was transferred automatically into a sample loop (100 $\upmu$L) with a two-port needle and a gentle He stream (approx. 0.5 mL min$^{-1}$). Seven sample gas pulses were introduced onto a Rt-Molsieve 5 Å  PLOT column (Restek from BGB Analytik; 30 m x 0.32 mm ID, 30 $\upmu$m film thickness) through switching of a 6-port valve connecting and disconnecting the sample loop with the column. The column was kept at 30 °C in the GasBench column compartment or at 2 ${}^{\circ }$C in an external thermostat. Helium was the carrier gas with a flow of approximately 1.5 mL $\min ^{-1}$ at the end of the column. Nafion membranes for water removal were installed before the 6-port valve and after the column. The post-column carrier gas flow was introduced into the mass spectrometer via the Conflo device.

$\updelta ^{18}$O values were determined from the peak areas of masses 32 and 34 versus reference gas pulses of $\mathrm {O}_{2}$ gas, which were introduced into the IRMS at the beginning of each chromatogram. Averaged $\updelta ^{18}$O values of three and seven injection peaks for GC/IRMS and GasBench/IRMS, respectively, are reported in per mil (‰  ± standard deviation). In contrast to our previously proposed GC/IRMS method, [24] we were able to perform multiple injections from a single vial due to the overpressure in the vial preventing air contamination thereby increasing statistical precision (see Section S2). The $\updelta ^{18}$O values of the reference gas (3500 mV) were calibrated against $\mathrm {O}_{2}$ peaks from outside air (30 m GC/IRMS) or diluted outside air (60 m GC/IRMS and GasBench/IRMS). Here, we assume a constant $\updelta ^{18}\mathrm {O}_{\text {air}}$ value of 23.88 ‰ [33] noting the reported variations of $\updelta ^{18}\text {O}_{\text {air}}$ between $23.4\permille$ and $24.2\permille$ [34–37]. To ensure accuracy of the measured $\updelta ^{18}$O values, three samples with repeated injections were followed by three injections of ambient air. Method detection limits (MDL) were determined as described in Jochmann et al.[38] assuming a measurement precision of 0.6‰ [39]. With GC/IRMS, triplicate injections of 16 to 660 nmol of $\mathrm {O}_{2}$ were made both with variable sample concentrations and variable injection volumes. For determining MDL in GasBench/IRMS measurements, seven injections of 1.8–17.8 nmol $\mathrm {O}_{2}$ were made from Exetainers containing different amounts of dilute air. The resulting MDLs were 280 $\upmu$M and 18 $\upmu$M gaseous O$_2$ concentrations for GC/IRMS and GasBench/IRMS, respectively, which corresponded to average peak heights of approximately 2700 mV and 500 mV, respectively.

### Data evaluation

#### Peak integration

Automatic peak integration in our GC/IRMS setup with the 30 m GC column (Isodat NT 3.0, Thermo Fisher Scientific) with and without modified peak detection parameters did not allow separation of Ar and $\mathrm {O}_{2}$ signals. Manual peak integration was carried out through visual inspection of the *m*/*z* 34/32 signal ratios (Figure S4). To correct raw O isotope signatures, $\updelta ^{18}$O$^\star$, after automatic peak integration for Ar interferences, we defined a linear correction function as in Eq. 1 to return corrected values ($\updelta ^{18}$O$_\text {corr})$. This function accounts for the relative Ar and $\mathrm {O}_{2}$ peak areas on the basis of concentration ratio of Ar and $\mathrm {O}_{2}$ in sample gases (i.e. $c_{\text {Ar}}/c_{\mathrm {O}_{2}}$ in the headspace of sample vials). Deviations of $\updelta ^{18}$O$^\star$ scale linearly with $c_{\text {Ar}}/c_{\mathrm {O}_{2}}$ based on the correction factor *b*, which was obtained from measuring $^{18}$O/$^{16}$O ratios of sample gases with different $c_{\text {Ar}}/c_{\mathrm {O}_{2}}$ ratios and a weighted linear least-square regression. Uncertainties correspond to 95% confidence intervals. $\updelta ^{18}$O values of $\mathrm {O}_{2}$ in samples that had been purged with synthetic air did not show any visible Ar peaks and were obtained by automatic peak integration.

1 $$\begin{aligned} \updelta ^{18}\text {O}_{\text {corr}} = \updelta ^{18}\text {O}^{\star } - b \cdot c_{\text {Ar}}/c_{\mathrm {O}_{2}} \end{aligned}$$

Note that the baseline separation of the Ar from the $\mathrm {O}_{2}$ peak interfered with the automatic individual background determination in Isodat 3.0. For measurements of $^{18}$O/$^{16}$O ratios on GC/IRMS instrumentation equipped with 60 m chromatographic columns, a time-based background determination 1 min before the $\mathrm {O}_{2}$ peak resulted in the most consistent $\updelta ^{18}$O values. For measurements of $^{18}$O/$^{16}$O ratios on GasBench/IRMS devices with columns cooled to 2 ${}^{\circ }$C, the “skimmed background” determination in Isodat 3.0 gave the most consistent results.

#### Blank correction

To account for diffuse contamination with ambient $\mathrm {O}_{2}$, blank samples consisted of $\mathrm {N}_{2}$ filled vials for gaseous samples or $\mathrm {N}_{2}$ purged water for aqueous samples and were freshly prepared and run with each sequence. According to established procedures, blank corrections of measured $\updelta ^{18}$O values, $\updelta ^{18}\text {O}_{\text {meas}}$, with and without corrections for Ar interferences were made as in Eq. 2 [40].

2 $$\begin{aligned} \updelta ^{18}\text {O} = \frac{{\updelta ^{18}\text {O}_{\text {meas}}\cdot {\text {A}_{\text {meas}}}-{\updelta ^{18}}\text {O}_{\text {blank}}\cdot {\text {A}_{\text {blank}}}}}{\text {A}_{\text {meas}}-{\text {A}_{\text {blank}}}} \end{aligned}$$

where $\updelta ^{18}\text {O}_{\text {blank}}$ is the $\updelta ^{18}$O of blank measurements and $\text {A}_{\text {meas}}$ and $\text {A}_{\text {blank}}$ are the peak areas of mass 32 of the sample and the blank, respectively.

#### Oxygen isotope enrichment factors and $^{18}$O kinetic isotope effects

Oxygen isotope fractionation of dissolved $\mathrm {O}_{2}$, $\upepsilon _{\text {O}}$, associated with the reduction of $\mathrm {O}_{2}$ were derived with Eq. 3 on the basis of $\updelta ^{18}$O values with and without correction for Ar interferences. Equation 3 was solved through weighted non-linear least-square regression and the reported uncertainties correspond to 95% confidence intervals [13]. The corresponding $^{18}$O kinetic isotope effects, $^{18}$O KIE, derive from $\upepsilon _{\text {O}}$ values as in Eq. 4.

3 $$\begin{aligned} \frac{{\updelta ^{18}\text {O}}+1}{{\updelta ^{18}\text {O}_{0}}+1}= & {} \left( \frac{c_{\mathrm {O}_{2},w}}{c_{\mathrm {O}_{2},w}^0}\right) ^{{\upepsilon _{\text {O}}}}\end{aligned}$$

4 $$\begin{aligned} {{}^{18}\text {O-KIE}}= & {} \frac{1}{1+{\upepsilon _{\text {O}}}} \end{aligned}$$

where $\updelta ^{18}\text {O}_{0}$ are initial $\updelta ^{18}$O values and $c_{\mathrm {O}_{2},w}/c_{\mathrm {O}_{2},w}^0$ is the fraction of remaining $\mathrm {O}_{2}$.

## Results and discussion

### Extent of Ar interferences on $\updelta ^{18}$O of $\mathrm {O}_{2}$ and correction procedures

We evaluated the effects of Ar interference on $^{18}$O/$^{16}$O ratio measurements of $\mathrm {O}_{2}$ on GC/IRMS and GasBench/IRMS instrumentation using a suite of gas samples containing variable amounts of $\mathrm {O}_{2}$ with different origin (i.e. ambient vs. synthetic air) and analysed the data according to different procedures (i.e. automatic vs. manual peak integration). We present a selection of the most important results in the following. A detailed survey of conditions, for which the effect of Ar interferences on $\updelta ^{18}$O was quantified, is shown in Tables S2 and S3.

#### Observations on different CF/IRMS instrumentation

**Fig. 1:**
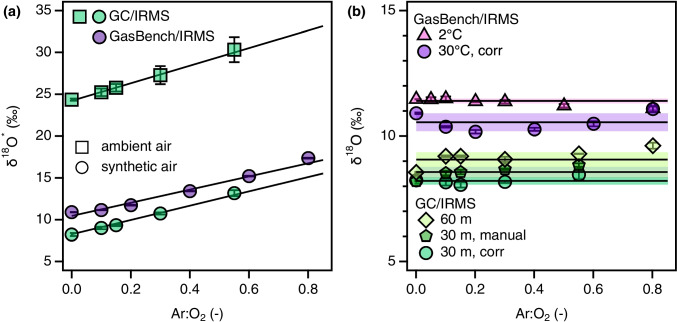
(**a**) $\updelta ^{18}$O$^\star$ values of $\mathrm {O}_{2}$ in gaseous samples of ambient and synthetic air prepared with different Ar:O_2_ ratios and measured by GC/IRMS and GasBench/IRMS. Note that different batches of synthetic air were used for sample preparation in GC/IRMS and GasBench/IRMS. Data correspond to entries 1–3, 10–12 in Table S2 and entry 2 in Table S3. (**b**) $\updelta ^{18}$O values after removal of Ar interferences by improved chromatographic Ar and $\mathrm {O}_{2}$ separation vs. $\updelta ^{18}$O values after applying different automated (“corr”) and “manual” correction procedures. Error bars and shaded areas represent ± one standard deviation, solid lines average values

When operating GC/IRMS and GasBench/IRMS devices with standard instrument parameters, that is with chromatography columns of typical length (30 m) at 30 ${}^\circ$C, we observed linear increases in $\updelta ^{18}$O$^{\star }$ with increasing Ar concentrations in the gas phase (Fig. 1). This finding was made with injections of ambient and synthetic air corresponding to amounts of injected $\mathrm {O}_{2}$ of 10.5 and 178 to 892 nmol of $\mathrm {O}_{2}$ with GasBench/IRMS and GC/MS, respectively (Tables S2 and S3, Figures S2 and S3). When averaged over the different amounts of injected $\mathrm {O}_{2}$, of $\updelta ^{18}$O vs. Ar:$\mathrm {O}_{2}$ ratios resulted in slopes *b* of $8.57\pm 0.16$ and $7.89\pm 0.09$ for GC/IRMS and GasBench/IRMS instruments, respectively (Tables S2 and S3). Despite the similar numbers, the extent of Ar interference quantified with slopes *b* is an instrument-specific number, determined, for example, by the degree of Ar and $\mathrm {O}_{2}$ separation [27, 28]. We found that *b* remained constant over at least one month ($9.24\pm 1.70$) in GC/IRMS setup, consistent with previous reports for $^{18}$O/$^{16}$O ratio measurements by DI/IRMS [26]. The correction factor increased only slightly ($11.0\pm 1.6$) after instrument reconfiguration five months later (Table S2). For accurate corrections, we therefore recommend reassessment of the Ar interference every month and after each reconfiguration although the expected changes in Ar interference are minor.

#### Correcting for and avoiding Ar interferences in CF-IRMS

The reproducible quantification of Ar interferences on $^{18}$O/$^{16}$O ratio measurements implies that post-measurement corrections of $\updelta ^{18}$O$^\star$ are feasible. Using the slopes *b* as correction factors in Eq. 1, one obtains constant, post-correction $\updelta ^{18}$O values of $8.2\pm 0.1\permille$ and $10.6\pm 0.4\permille$ for $\mathrm {O}_{2}$ measured in synthetic air and standard conditions (30 m at 30$^{\circ }$C) by GC/IRMS and GasBench/IRMS, respectively (Fig. 1b, “corr”). Note that different batches of synthetic air were used for the GC/IRMS and GasBench/IRMS samples and, hence, $\updelta ^{18}$O do not refer to identical $\mathrm {O}_{2}$ specimen. While we applied a correction factor that was derived from a series of artificial gas samples with known Ar:$\mathrm {O}_{2}$ ratios, the same type of correction can be made without such additional calibrations. As shown in Figure S2d, Ar:$\mathrm {O}_{2}$ ratios can also be obtained reliably from *m/z* 40 and *m/z* 32 peak areas ratios. Finally, we note that in cases where derivation of correction factors *b* from the correlation of $\updelta ^{18}$O vs. Ar:$\mathrm {O}_{2}$ ratios is impractical, Ar interference can also be excluded reasonably well by manual integration of the $\mathrm {O}_{2}$ peak after the Ar peak (Section S4). An example for this procedure with the identical samples of synthetic air analysed by GC/IRMS is shown in Fig. 1b. This approach is somewhat arbitrary but the outcome can nevertheless lead to $\updelta ^{18}$O values that are comparable to those obtained by automatic data evaluation procedures ($8.57\pm 0.23$‰).

If the same type of samples are measured by GC/IRMS equipped with a 60 m molsieve column, we obtain baseline separated Ar and $\mathrm {O}_{2}$ peaks and the $\updelta ^{18}$O values at different Ar:$\mathrm {O}_{2}$ ratios amount to $9.1\pm 0.3\permille$ (Fig. 1b). On a GasBench/IRMS, a complete separation of Ar and $\mathrm {O}_{2}$ peaks by cooling the column to 2$^{\circ }$C results in $\updelta ^{18}$O values that agree with those obtained through correction ($11.4\pm 0.1\permille$). The quality of separation in the 2$^{\circ }$C GasBench/IRMS setup remained constant over several months and reconfigurations (Table S3, Figure S3). While the samples analysed with and without improved chromatography are not identical, we find that the differences in $\updelta ^{18}$O within data for each GC/IRMS and GasBench/IRMS is below the value of $\pm 0.6\permille$ suggested as total uncertainty for $^{18}$O/$^{16}$O measurements by CF-IRMS [41].

### Consequences of Ar interference for quantification of O isotope fractionation and interpretation of $^{18}$O kinetic isotope effects

In laboratory experiments where changes of $\updelta ^{18}$O are correlated with the fractional amount of conversion to derive O isotope fractionation factors $\upepsilon _{\mathrm {O}}$ and $^{18}$O KIEs as in Eq. 3, Ar interferences can potentially bias the quantification of these parameters. The observed $\updelta ^{18}$O shift towards higher values (Fig. 1) amplifies the preference for reactions of $^{16}$O isotopologues of $\mathrm {O}_{2}$ and results in overestimated isotope fractionation and larger $^{18}$O KIEs. Conversely, inverse isotope effects that generate smaller $\updelta ^{18}$O due to preferred reactions of $^{18}$O isotopologues, [42–44] could be disguised, especially, at early states of $\mathrm {O}_{2}$ conversion.

#### Theoretical considerations

We studied the theoretical consequences of Ar interferences on the quantification of $\upepsilon _{\mathrm {O}}$ values by assuming that the measured $\updelta ^{18}$O not only reflect changes due to isotope fractionation from kinetic isotope effects but also from Ar interferences. To that end, Eq. 3, which describes changes of $\updelta ^{18}$O from O isotope fractionation, was complemented to include contributions from Ar interferences to $\updelta ^{18}$O as term $b \cdot c_\text {Ar}/c_{\mathrm {O}_{2}}$ from Eq. 1 to form Eq. 5. As shown in the Sections S5 and S6, the contributions of Ar inferences to $\updelta ^{18}$O$^*$ can be expressed as a function of the reaction progress (i.e. $c_{\mathrm {O}_{2},w}/c_{\mathrm {O}_{2},w}^0$) using a constant, $\mathcal {P}$, that describes the partitioning of Ar and $\mathrm {O}_{2}$ into the headspace of the sample vials (Eq. S12) to form Eq. 6.

5 $$\begin{aligned} \delta ^{18}\mathrm {O}^{\star }= & {} \left( (\delta ^{18}\text {O}_0 + 1 ) \cdot (c_{\mathrm {O}_{2},w}/c_{\mathrm {O}_{2},w}^0)^{{\upepsilon }_\text {O}} - 1 \right) + b \cdot c_\text {Ar}/c_{\mathrm {O}_{2}} \end{aligned}$$

6 $$\begin{aligned}= & {} \left( (\delta ^{18}\text {O}_0 + 1 ) \cdot (c_{\mathrm {O}_{2},w}/c_{\mathrm {O}_{2},w}^0)^{{\upepsilon }_\text {O}} - 1 \right) + b \cdot \mathcal {P}/\left( c_{\mathrm {O}_{2},w}^0 \cdot c_{\mathrm {O}_{2},w}/c_{\mathrm {O}_{2},w}^0 \right) \end{aligned}$$

where superscript ${}^{\star }$ stands for measured and uncorrected $\updelta ^{18}$O values of $\mathrm {O}_{2}$ and $\updelta ^{18}$O$_0$ is its initial, true value.

Insertion of Eqs. 6 into 4 allows for an expression of the observable $\upepsilon _{\mathrm {O}}^{\star }$ from uncorrected $\updelta ^{18}$O$^\star$ values that can be compared to the “true” $\upepsilon _{\mathrm {O}}$ in Eq. 7 (see Section S6 for derivation).

7 $$\begin{aligned} \Delta {\upepsilon _{\mathrm {O}}} = {\upepsilon _{\mathrm {O}}} - {\upepsilon _{\mathrm {O}}^{\star }} \end{aligned}$$

**Fig. 2:**
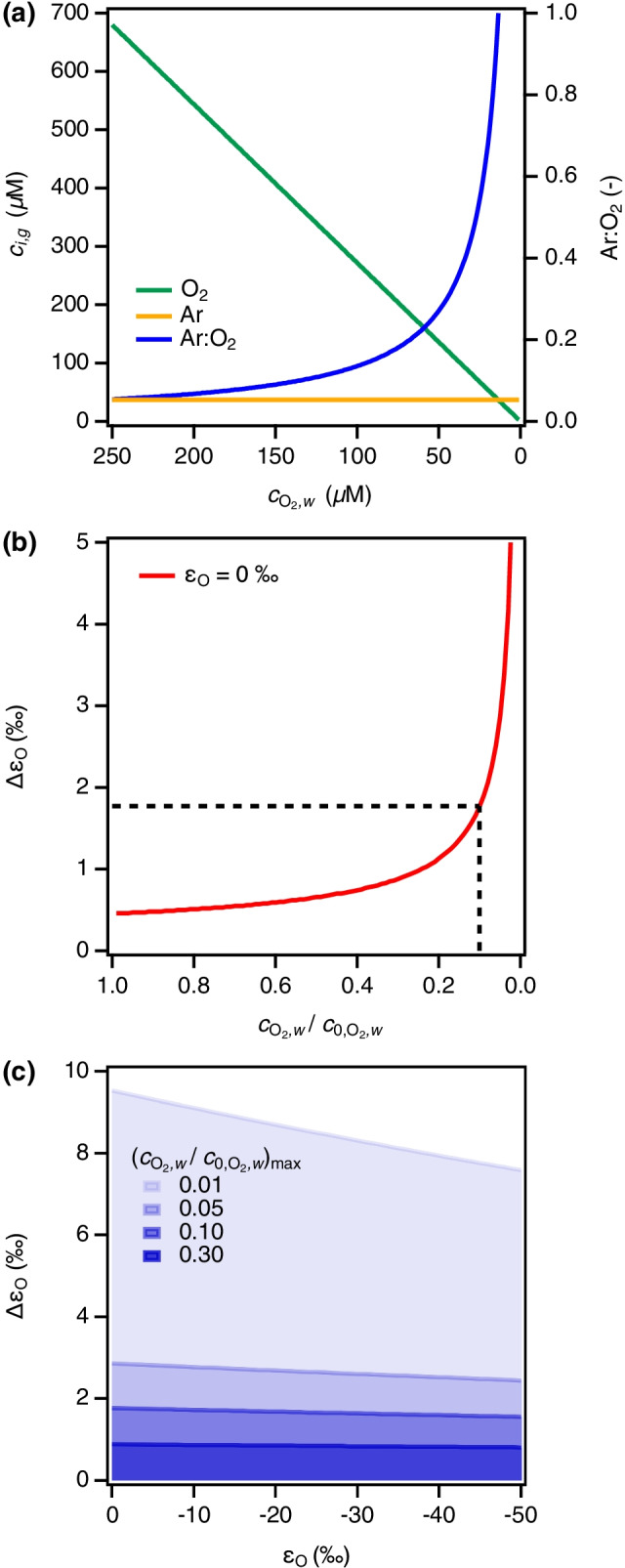
(**a**) Theoretical experiment representing the removal of $\mathrm {O}_{2}$ from an aqueous solution with concomitant increase of Ar:$\mathrm {O}_{2}$ ratio. The gas phase $\mathrm {O}_{2}$ concentration, $c_{i,g}$, corresponds to the extraction of 250 $\upmu$M O_2_ from 9 mL aqueous solution into a 3 mL headspace (see Section S5 for details). (**b**) Apparent O isotope fractionation of $\mathrm {O}_{2}$ caused by Ar interferences only. $\Delta \upepsilon _{\mathrm {O}}$ stands for the difference between the apparent enrichment factor $\epsilon _{\mathrm {O}}^{\star }$ and the “true” value of $0\permille$. The dashed line illustrates the $\Delta \upepsilon _{\mathrm {O}}$ for a reaction conversion of 90%. (**c**) $\Delta \upepsilon _{\mathrm {O}}$ determined for different theoretical $\upepsilon _{\mathrm {O}}$ and variable extents of reactant conversion on the basis of Eq. S2

We systematically evaluated the deviation of uncorrected $$\upepsilon _{\mathrm {O}}^{\star }$$ from $$\upepsilon _{\mathrm {O}}$$ as $$\Delta \upepsilon _{\mathrm {O}}$$  (Eq. 7) for a theoretical experiment in which 250 $$\upmu$$M of dissolved $$\mathrm {O}_{2}$$ are consumed in the presence of a constant Ar background of 14 $$\upmu$$M (Fig. 2a). In the absence of any reaction-related O isotope fractionation of $$\mathrm {O}_{2}$$ (i.e. $$\upepsilon _{\mathrm {O}}$$ $$= 0\permille$$, Fig. 2b), the increasing Ar:$$\mathrm {O}_{2}$$ ratio in the headspace causes $$\Delta$$$$\upepsilon _{\mathrm {O}}$$ to increase dramatically with increasing fractional $$\mathrm {O}_{2}$$ conversion. Choosing an arbitrary but realistic scenario of $$90\%$$ reactant conversion with considerable Ar interferences ($$c_\text {Ar}/c_{\mathrm {O}_{2}}$$ of approx. 0.5 corresponding to $$4\permille$$ deviation in $$\updelta ^{18}\text {O}^\star$$ based on Eqs. S12 and S16, respectively) for calculation of $$\updelta ^{18}\text {O}^\star$$, we find that $$\Delta \upepsilon _{\mathrm {O}}$$ derived with Eq. S21 lead to an overestimation of $$\upepsilon _{\mathrm {O}}$$ by $$1.8\permille$$ (Fig. 2b). $$\Delta \upepsilon _{\mathrm {O}}$$ decreases slightly with increasing extent of O isotope fractionation (i.e. more negative $$\upepsilon _{\mathrm {O}}$$ values) over an $$\upepsilon _{\mathrm {O}}$$-range that corresponds to $${}^{18}$$O KIE between 1.003 to 1.040 reported for typical biological and abiotic $$\mathrm {O}_{2}$$ reduction reactions [12, 14, 45]. Figure 2c illustrates how the bias of $$\upepsilon _{\mathrm {O}}$$ by Ar interferences varies with different extents of $$\mathrm {O}_{2}$$ conversion. If $$^{18}$$O/$$^{16}$$O ratio measurements are confined to only $$70\%$$ of $$\mathrm {O}_{2}$$ conversion ($$c_{\mathrm {O}_{2},w}/c_{\mathrm {O}_{2},w}^0 = 0.3$$), $$\Delta \upepsilon _{\mathrm {O}}$$ is $$<1\permille$$. Such boundary conditions for quantification of $$\upepsilon _{\mathrm {O}}$$ could arise, for example, as a consequence of higher MDLs for $$^{18}$$O/$$^{16}$$O ratio analysis of $$\mathrm {O}_{2}$$ by GC/IRMS compared to GasBench/IRMS. While lower extent of reactant conversion might appear “favourable” to minimize Ar interferences, such situations typically increase the uncertainty of quantification of isotope enrichment factors and KIEs [46].

#### Experimental relevance

**Fig. 3:**
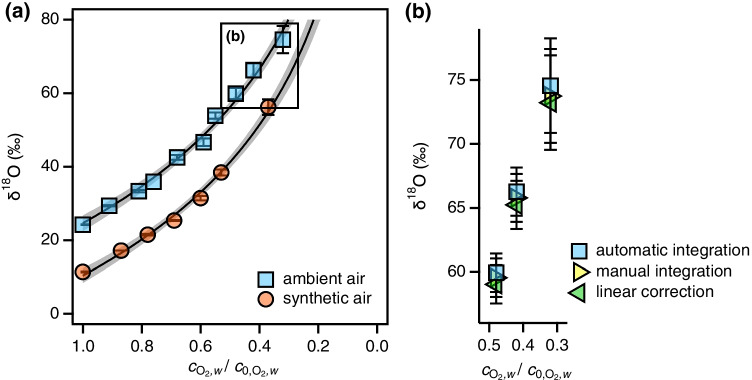
(**a**) $\updelta ^{18}$O of remaining $\mathrm {O}_{2}$ during reduction in glucose oxidase assays vs fraction of remaining dissolved $\mathrm {O}_{2}$, $c_{\mathrm {O}_{2},w}/c_{\mathrm {O}_{2},w}^0$, in buffer solutions saturated with ambient and synthetic air, respectively. Solid lines are fits to Eq. 3 with $\upepsilon _{\mathrm {O}}$ values shown in Table 1. Error bars represent standard deviations of triplicate measurements. Shaded areas represent $95\%$ confidence intervals. Data for the rectangular inset is shown in panel (**b**). (**b**) $\updelta ^{18}$O values from panel (**a**) obtained with different data treatment procedures

We examined the consequences of Ar interferences on the experimental determination of $\upepsilon _{\mathrm {O}}$ and $^{18}$O KIEs in enzymatic $\mathrm {O}_{2}$ reduction experiments with glucose oxidase measured on GC/IRMS. The decrease of $\mathrm {O}_{2}$ concentrations by 0.81 and 0.69 $\upmu$M $\mathrm {O}_{2}$ per $\upmu$M of glucose in Ar-saturated and Ar-free buffer, respectively, confirmed that $\mathrm {O}_{2}$ was reductively transformed (see Section S7). Figure 3a shows the substantial O isotope fractionation of $\mathrm {O}_{2}$ measured in experiments with air-saturated buffer solution containing approximately 14 $\upmu$M Ar vs. buffer solutions purged with synthetic air that did not contain any Ar. While $\mathrm {O}_{2}$ in synthetic air exhibits a lower $\delta ^{18}$O than ambient air (8–$12 \permille$ vs. $23.88 \permille$ [33], Fig. 1a), both O isotope fraction trends appear largely identical. Note that due to the moderately high MDL of $\mathrm {O}_{2}$ for the GC/IRMS instrument setup (Section S2), the extent of $\mathrm {O}_{2}$ conversion was limited to $68\%$. These experimental conditions limited the Ar:$\mathrm {O}_{2}$ ratio to 0.15. As a consequence, overestimation of $\updelta ^{18}$O is relatively minor (0.6–$1.9\permille$) and within the uncertainty of triplicate measurements (Fig. 3b). In agreement with data shown in Fig. 1a, uncorrected $\updelta ^{18}$O exceeded those after correcting for Ar interferences and through manual peak integration.

$\upepsilon _{\mathrm {O}}$ and $^{18}$O KIEs for $\mathrm {O}_{2}$ reduction were high and amounted to $-43\permille$ and 1.045, respectively (Table 1). $\upepsilon _{\mathrm {O}}$ and $^{18}$O KIE values derived from samples with and without Ar interferences (i.e. from solutions with ambient vs. synthetic air) show consistent trends but the observed differences in $\upepsilon _{\mathrm {O}}$ of $\le 1\permille$ (≤0.001 KIE units) are not significant, that is, the numbers vary within the statistical uncertainty of $3\permille$ (Table 1). For experiments in ambient-air purged solutions, (linear) correction for Ar interferences led to smaller $\upepsilon _{\mathrm {O}}$. The $\upepsilon _{\mathrm {O}}$ value corrected for Ar interferences was indeed identical to the one measured for experiments in solutions prepared with synthetic air. Our experimental data corroborate the theoretical findings (Eq. S21) which predict a $\Delta \upepsilon _{\mathrm {O}}$ of $0.76\permille$ for $c_{\mathrm {O}_{2},w}/c^0_{\mathrm {O}_{2},w}$ of 0.32 and an $\upepsilon _{\mathrm {O}}$ of $-43.2\permille$. Despite a large number of data points for the derivation of $\upepsilon _{\mathrm {O}}$ and $^{18}$O KIEs, their experimental uncertainty exceeds any effect of Ar interferences on measured $^{18}$O/$^{16}$O ratios and $\updelta ^{18}$O values. With GasBench/IRMS, the MDL is significantly lower and Ar interference may have been more pronounced depending on the maximum turnover in samples. Section S8 includes a discussion of the same experiment with single injections and a lower MDL as described previously [24].

**Table 1:** Oxygen isotope enrichment factors, $\upepsilon _{\text {O}}$, and ${}^{18}$O kinetic isotope effects, ${}^{18}$O-KIE, of $\mathrm {O}_{2}$ reduction by glucose oxidase in experiments with aqueous solutions purged with ambient vs. synthetic air$^{\mathrm {a,b}}$

Data treatment	$\upepsilon _{\mathrm {O}}$ (‰)	${}^{18}$O-KIE (-)
*Ambient air*		
Automatic	$-43.9\pm 3.4$	1.046 ± 0.004
Manual	$-43.3\pm 3.5$	1.045 ± 0.004
Linear correction	$-42.9\pm 3.5$	1.045 ± 0.004
*Synthetic air*		
Automatic	$-43.2 \pm 3.5$	1.045 ± 0.004

${}^{\mathrm {a}}$ $\upepsilon _{\mathrm {O}}$ and $^{18}$O-KIE from experiments with ambient air were corrected for Ar interferences with different data treatment procedures after $^{18}$O/$^{16}$O ratio measurements on a GC/IRMS equipped with a 30 m cloumn.

${}^{\mathrm {b}}$ Uncertainties represent 95% confidence intervals

## Conclusions

Our data illustrate that $^{18}$O/$^{16}$O ratio measurements in $\mathrm {O}_{2}$ by CF-IRMS (GC/IRMS and GasBench/IRMS) are subject to Ar interferences that lead to increases of $\updelta ^{18}$O values by up to $6\permille$. Depending on instrument availability, users can resolve these issues with simple modifications of the instrumental setup that lead to an improved chromatographic separation of Ar and $\mathrm {O}_{2}$ signals in the isotope ratio mass spectrometer. Alternatively, linear corrections of $\updelta ^{18}$O through quantification of the isotope signature bias vs. Ar:$\mathrm {O}_{2}$ ratios offer a straightforward option for post-measurement corrections.

We find that Ar interferences have largest effects on $\delta ^{18}$O in samples, where $\mathrm {O}_{2}$ has been consumed through reactive processes such as enzymatic or chemical reduction. This phenomenon leads to an overestimation of O isotope fractionation in these processes and, ultimately, to larger $^{18}$O KIE and more negative $\upepsilon _{\mathrm {O}}$ values. As confirmed experimentally, this effect is confined to $<2\permille$ in $\upepsilon _{\mathrm {O}}$ and typically smaller than other sources of uncertainty such as the precision of repeated $^{18}$O/$^{16}$O ratio measurements as well as $\upepsilon _{\mathrm {O}}$ variability between replicate experiments. Consequences of Ar interference on $\updelta ^{18}$O, however, increase more strongly once the fractional amount of $\mathrm {O}_{2}$ conversion exceeds $90\%$, that is below approximately 25 $\upmu$M of residual $\mathrm {O}_{2}$ in aqueous solution at ambient temperature. Such fractional amounts of $\mathrm {O}_{2}$ conversion are critical, especially for accurate quantification of small extent of O isotope fractionation. The relatively high MDLs of some CF-IRMS methods such as the GC/IRMS setup used here for multiple gas injections from headspace samples will make $^{18}$O/$^{16}$O ratio measurements in this regime impossible and Ar interferences of minor relevance. Quantification of O isotope fractionation with other instrumental procedures such as single injection GC/IRMS [24] or GasBench/IRMS, by contrast, requires measures for eliminating Ar interferences as evaluated and proposed in this study.

## Electronic supplementary material

Below is the link to the electronic supplementary material.

Supplementary file1 (PDF 740 KB)

## Data Availability

List of materials used, evaluation of GC/IRMS setup for repeated headspace sample injections from the same sample, material, survey of experiments on Ar interference, equations for the derivation of the theoretical extent of overestimation of O isotope fractionation, and additional data for experiments with glucose oxidase.
