# Supplementary material for: Managing argon interference during measurements of 18O/16O ratios in O2 by continuous-flow isotope ratio mass spectrometry
Source: Anal Bioanal Chem. 2022 Jul 16;414(20):6177–86. doi: 10.1007/s00216-022-04184-3 (PMC9314310; doi:10.1007/s00216-022-04184-3)
Supplement: Supplementary file 1 — Supplementary file1 (PDF 740 KB) [file 216_2022_4184_MOESM1_ESM.pdf]

Supplementary Information for
Analytial and Bioanalytical Chemistry

Managing Argon Interference during Measurements of $^{18}O/^{16}O$ Ratios in O_2 by Continuous-Flow Isotope Ratio Mass Spectrometry

Charlotte E. Bopp,[1,2] Jakov Bolotin,[1] Sarah G. Pati,[*,3] and Thomas B. Hofstetter[*,1,2]

[1]Eawag, Swiss Federal Institute of Aquatic Science and Technology, CH-8600 Dübendorf, Switzerland, [2]Institute of Biogeochemistry and Pollutant Dynamics (IBP), ETH Zürich, CH-8092 Zürich, Switzerland, [3]Department of Environmental Sciences, University of Basel, CH-4056 Basel, Switzerland

*Corresponding authors:
sarah.pati@unibas.ch and
thomas.hofstetter@eawag.ch

17 Pages, 6 Figures, 3 Tables

Contents

S1 Materials **3**

S2 Modified GC/IRMS setup for repeated headspace sample injections **3**
S2.1 Derivation of the maximum number of gas injections 3

S3 Extent of Argon Interference **5**
S3.1 Previous reports for DI/IRMS 5
S3.2 This study for GC/IRMS and GasBench/IRMS 6

S4 Manual peak integration **11**

S5 Quantification of Ar:O_2 ratios **11**

S6 Consequences of Ar interferences on O_2 **12**
S6.1 Derivation of theoretical relationship 12
S6.2 Illustrative calculations 14

S7 O_2 reduction by glucose oxidase **16**

S8 Estimation of Argon interference at low MDLs **16**

S9 References **17**

S1 Materials

Argon (99.999%), N_2 (99.999%), O_2 (99.995%), He (99.999%), and synthetic air (20% O_2, 80% N_2) were obtained from Carbagas (Gümligen, Switzerland). All chemicals and enzymes were purchased from Sigma-Aldrich (Buchs, Switzerland) and used as received. Sodium acetate buffer was prepared with sodium acetate (99%) and hydrochloric acid (HCl, 32%). For O_2 consumption experiments, we used D-(+)-glucose (99.5%), and glucose oxidase from Aspergillus niger (type VII, 224 890 units g^{-1}). All solutions were prepared in ultrapurified water (18.2 MΩ·cm, NANOpure Diamond water purification system; Barnstead). O_2-free solutions were obtained by heating water to 90 °C for 30 min while purging with N_2 gas. Purging continued thereafter for at least 2h. Samples containing O_2-free water were prepared in an anaerobic glove box (< 1 ppm O_2) with a N_2 atmosphere (Unilab 2010; MBraun GmbH, Germany).

S2 Modified GC/IRMS setup for repeated headspace sample injections

We modified our instrumental procedures for $^{18}O/^{16}O$ measurements by GC/IRMS compared to our previous work[1] to allow for multiple, automated gas injections from the same headspace sample. This approach is useful if the number of samples from O isotope fractionation experiments and the aqueous sample volumes are limited.[2] The modified procedure allows for increasing the statistical precision of $\delta^{18}O$ values and thus the ε_O values used for the quantification of O isotope fractionation of O_2 reduction. As shown in detail in the following, the modified procedure involved increasing the pressure of the headspace in the sample vials. This measure resulted in a dilution of the O_2 content of the samples, a consequent increase of the method detection limit (MDL), and the need to increase the sample injection volume. Here, we evaluated signals areas at m/z 32 exceeding 10 Vs, corresponding to approximately 120 nmol O_2 injected. With the parameters described in the method section, this corresponds to a MDL of 280 µM O_2 in the headspace and 100 µM dissolved O_2 in aqueous samples, respectively, for triplicate injections of 1000 μM. The MDL was thus approximately 6 times higher than reported for for the singe-injection setup (16 µM final aqueous O_2 concentration).[1] In the following, we show that by introducing N_2 gas to the sample vials to a pressure of 2 bars, the number of possible injections from sample headspace into a GC/IRMS could, theoretically, be increased to up to 16.

S2.1 Derivation of the maximum number of gas injections from the sample head space

Multiple injections from a single vial are possible if there is sufficient overpressure inside the vials. This can be rationalised based on the assumption of ideal gases and the ideal gas law (eq.

S1).

$$pV = nRT \tag{S1}$$

where p is the pressure (bar), V is the volume (L), n is the amount of substance (µmol), R is the ideal gas constant, 8.314 J K^{-1} mol^{-1}, and T is the absolute temperature. We assume that sample vials are tight and retain a constant pressure even after multiple piercings of the septa. Each injection, x, reduces the amount of O_2, $n_{O_2}(x)$, and the pressure inside the vial, $p_{\text{vial}}(x)$, by the amount removed by the syringe according to equations S2 to S5.

$$\frac{dn_{O_2}}{dx} = -n_{O_2}(x) \cdot \frac{V_{\text{inj}}}{V_{\text{inj}} + V_{\text{vial}}} \tag{S2}$$

$$n_{O_2}(x) = n^0_{O_2} \cdot e^{-\frac{V_{\text{inj}}}{V_{\text{inj}}+V_{\text{vial}}} \cdot x} \tag{S3}$$

$$\frac{dp_{\text{vial}}}{dx} = -p_{\text{vial}}(x) \cdot \frac{V_{\text{inj}}}{V_{\text{inj}} + V_{\text{vial}}} \tag{S4}$$

$$p_{\text{vial}}(x) = p^0_{\text{vial}} \cdot e^{-\frac{V_{\text{inj}}}{V_{\text{inj}}+V_{\text{vial}}} \cdot x} \tag{S5}$$

where V_{inj} is the injection volume, V_{vial} is the effective gaseous volume of the vial, and $n^0_{O_2}$ and p^0_{vial} are the initial values of n_{O_2} and p_{vial}, respectively. Figure S1(a) illustrates how both n_{O_2} and p_{vial} decay by the same rate. The amount of O_2 withdrawn in the syringe is reduced accordingly. When the syringe is removed from the vial, however, pressure equalises to ambient pressure, p_{amb}, with a loss of sample proportional to the decreasing difference in pressure. As long as there is overpressure inside the vial, i.e. $p_{\text{vial}}(x) > p_{\text{amb}}$, the amount of O_2 injected into the IRMS, $n^{\text{inj}}_{O_2}$, is given by equation S6.

$$n^{\text{inj}}_{O_2}(x) = \frac{p_{\text{amb}}}{p_{\text{vial}}(x)} \cdot \frac{n_{O_2}(x) \cdot V_{\text{inj}}}{V_{\text{inj}} + V_{\text{vial}}} = \frac{p_{\text{amb}}}{p^0_{\text{vial}}} \cdot \frac{n^0_{O_2} \cdot V_{\text{inj}}}{V_{\text{inj}} + V_{\text{vial}}}, \text{ where } p_{\text{vial}}(x) > p_{\text{amb}} \tag{S6}$$

Thus, $n^{\text{inj}}_{O_2}$ is constant for as long as sample is escaping the syringe for pressure equalisation (Fig S1b). O_2 contamination from ambient air is limited to diffusive contamination into the syringe during sample transfer to the injector. Once $p_{\text{vial}}(x)$ approaches p_{amb}, the amount of sample O_2 in the syringe is limited by the residual O_2 concentration in the vial and contaminated by ambient air containing χ vol% O_2 entering the syringe during pressure equalisation according to equation S7.

$$n^{\text{inj}}_{O_2}(x) = \frac{p_{\text{amb}} - p_{\text{vial}}(x)}{R \cdot T} \cdot V_{\text{inj}} \cdot \chi + \frac{n_{O_2}(x) \cdot V_{\text{inj}}}{V_{\text{inj}} + V_{\text{vial}}}, \text{ where } p_{\text{vial}}(x) \leq p_{\text{amb}} \tag{S7}$$

Consequently, the maximum number of injections, x_{max}, for reproducible measurements can be estimated by equation S8.

$$x_{\text{max}} = -\frac{(V_{\text{inj}} + V_{\text{vial}})}{V_{\text{inj}}} \cdot \ln\left(\frac{p_{\text{amb}}}{p^0_{\text{vial}}}\right) \tag{S8}$$

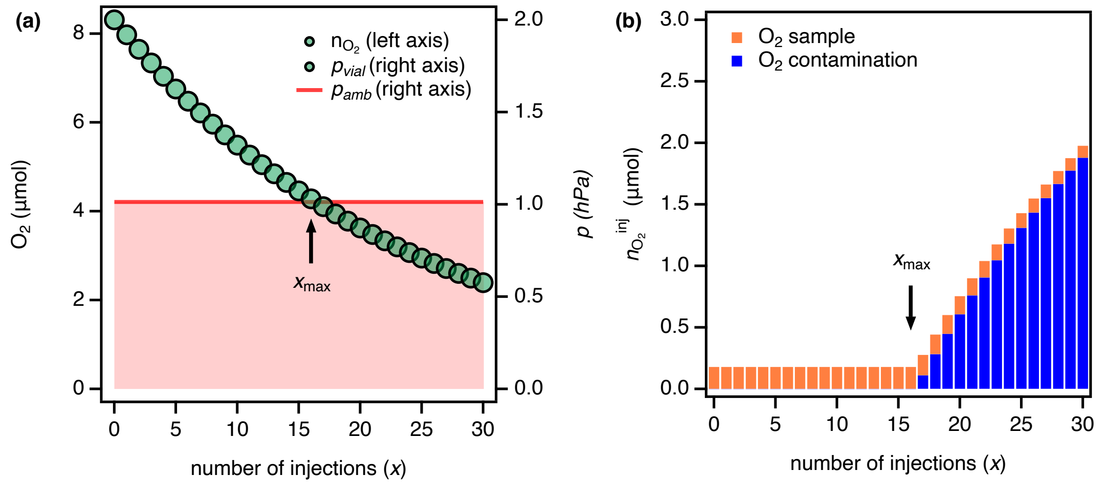

Figure S1 Example for predicted dynamics of total pressure and total O_2 inside sample vials (a) and of O_2 injected (b). Both graphs are based on parameters as described in the methods section. The initial amount of O_2 in the vial, $n^0_{O_2}$, is 8.31 µmol, which corresponds to 1 mL of artificial air in a 11.8 mL vial (V_{vial}), the initial pressure inside the vial, p^0_{vial}, is 2000 hPa, and each injection, x, is 500 µL (V_{inj}). Ambient pressure, p_{amb} is 1013 hPa and 20.9 vol% O_2 (χ). The maximum number of injections without significant air contamination, x_{max}, is indicated by an arrow.

The maximum injection volume or minimal vial pressure can be determined accordingly. To account for setup specific contamination, the minimal vial pressure can be tested by repeated injections from the same vial with a defined pressure. Once the amplitude of the O_2 peak increases, the maximum number of injections is reached.

S3 Extent of Argon Interference

S3.1 Previous reports for DI/IRMS

Argon interferences during measurements of $^{18}O/^{16}O$ and $^{17}O/^{16}O$ ratios of O_2 have been reported for analyses by dual-inlet isotope ratio mass spectrometry (DI/IRMS) and these studies are compiled in table S1. Linear correction factors for Ar interferences in ‰/Ar:O_2 for $\delta^{18}O$ and $\delta^{17}O$ according to eq. 1 of the main manuscript varied by several orders of magnitude. These values serve as a benchmark for the magnitude of Ar interference and underscore the need for instrument- and setup-specific calibration of Ar interference for O isotope mass spectrometry.

Table S1 Ar interference reported as correction factors b on measurements of $^{18}O/^{16}O$ and $^{17}O/^{16}O$ ratios of O_2 reported for analyses by dual-inlet isotope ratio mass spectrometry.

**Reference**	**b (‰/Ar:O_2)**		**Ar:O_2 basis**
	$\delta^{17}O$	$\delta^{18}O$	
Barkan and Luz [3]	0.0002747	0.0002210	m/z 40/32
Abe and Yoshida [4]	383[a,b]	128[a,c]	vol%
Sarma et al. [5]	5	1	m/z 40/32
Jurikova et al. [6]	0.01	-0.02	m/z 40/32

[a] approximate value from published figure [b] as $\delta^{33}O$ [c] as $\delta^{34}O$

S3.2 This study for GC/IRMS and GasBench/IRMS

Here, we assessed the consequences of Ar interference for the determination of $\delta^{18}O$ values at various O_2 concentrations for two types of instrumentation (GC/IRMS, GasBench/IRMS), two types of air samples, different chromatographic conditions, a range of injected O_2 masses, as well as over different time periods of instrument operation. The complete study data is compiled in Tables S2 and S3 for GC/IRMS, and GasBench/IRMS, respectively, which also includes references to the presentation of this data in different figures of the main manuscript and in this Supplementary Information (Figures S2 and S3).

Table S2 Compilation of Ar interference correction factor, b, for GC/IRMS measurements based on linear correlations of O_2 isotope signatures with ratios of Ar:O_2 in experiments with different chromatography strageties and amounts of analyte gas injected.

#	Instrument	Column / temperature	Peak integration	n_{O_2} (nmol)	O isotope ratio	number of injections	Correction factor b^a (‰/Ar:O_2)	Air source	Figure
1	GC/IRMS	30 m, 30 °C	automatic	178	$^{18}O/^{16}O$	3	9.77±1.01	synthetic	
2	GC/IRMS	30 m, 30 °C	automatic	446	$^{18}O/^{16}O$	3	8.19±0.37	synthetic	
3	GC/IRMS	30 m, 30 °C	automatic	892	$^{18}O/^{16}O$	3	8.84±0.52	synthetic	
1-3						9	8.57±0.16		1a, S2a
4[b]	GC/IRMS	30 m, 30 °C	automatic	178	$^{18}O/^{16}O$	1	9.99±1.32	synthetic	
5[b]	GC/IRMS	30 m, 30 °C	automatic	446	$^{18}O/^{16}O$	1	7.89±1.03	synthetic	
6[b]	GC/IRMS	30 m, 30 °C	automatic	892	$^{18}O/^{16}O$	1	9.86±1.44	synthetic	
4-6						3	9.24±1.70		S2b
7[c]	GC/IRMS	30 m, 30 °C	automatic	178	$^{18}O/^{16}O$	3	8.49±0.95	synthetic	
8[c]	GC/IRMS	30 m, 30 °C	automatic	446	$^{18}O/^{16}O$	3	12.2±0.5	synthetic	
9[c]	GC/IRMS	30 m, 30 °C	automatic	892	$^{18}O/^{16}O$	3	11.5±0.9	synthetic	
7-9						9	11.0±1.6		S2c
10[c]	GC/IRMS	30 m, 30 °C	automatic	178	$^{18}O/^{16}O$	3	6.78±0.63	ambient	
11[c]	GC/IRMS	30 m, 30 °C	automatic	446	$^{18}O/^{16}O$	3	12.4±1.1	ambient	
12[c]	GC/IRMS	30 m, 30 °C	automatic	892	$^{18}O/^{16}O$	3	12.3±0.4	ambient	
10-12						9	10.5±2.7		1a, S2c
13	GC/IRMS	30 m, 30 °C	manual	446	$^{18}O/^{16}O$	3	1.43±0.26	synthetic	1b
14[c]	GC/IRMS	60 m, 30 °C	tbBGD[d]	446	$^{18}O/^{16}O$	3	0.99±0.10	synthetic	1b

[a] Errors correspond to 95% confidence intervals; [b] measurement conducted one month after entries 1 to 3 of this table;
[c] measurement conducted five months after entries 1 to 3 of this table following instrument reconfiguration; [d] time based background.

Table S3 Compilation of Ar interference correction factor, b, for GasBench/IRMS measurements based on linear correlations of O_2 isotope signatures with ratios of Ar:O_2 in experiments with different chromatography strageties and amounts of analyte gas injected.

#	Instrument	Column / temperature	Peak integration	n_{O_2} (nmol)	O isotope ratio	number of injections	Correction[a] factor b (‰/Ar:O_2)	Air source	Figure
1	GasBench/IRMS	30 m, 30 °C	automatic	3.5	$^{18}O/^{16}O$	7	6.48±0.31	ambient	S3a
2	GasBench/IRMS	30 m, 30 °C	automatic	10.5	$^{18}O/^{16}O$	7	7.89±0.09	synthetic	1a, S3a
3	GasBench/IRMS	30 m, 2 °C	skmdBGD[b]	10.5	$^{18}O/^{16}O$	7	−0.49±0.18	synthetic	1b
4[c]	GasBench/IRMS	30 m, 2 °C	skmdBGD	3.5	$^{18}O/^{16}O$	7	−0.56±1	ambient	
5[c]	GasBench/IRMS	30 m, 2 °C	skmdBGD	7.0	$^{18}O/^{16}O$	7	−0.85±0.81	ambient	
6[c]	GasBench/IRMS	30 m, 2 °C	skmdBGD	10.5	$^{18}O/^{16}O$	7	−1.24±0.57	ambient	
7[c]	GasBench/IRMS	30 m, 2 °C	skmdBGD	14.0	$^{18}O/^{16}O$	7	−1.25±0.67	ambient	
5-7[d]						21	−1.16±0.29		S3b

[a] Errors correspond to 95% confidence intervals; [b] skimmed background [c] measurement conducted nine months after entry 3 of this table following instrument reconfiguration; [d] entry 4 was omitted due to MDL.

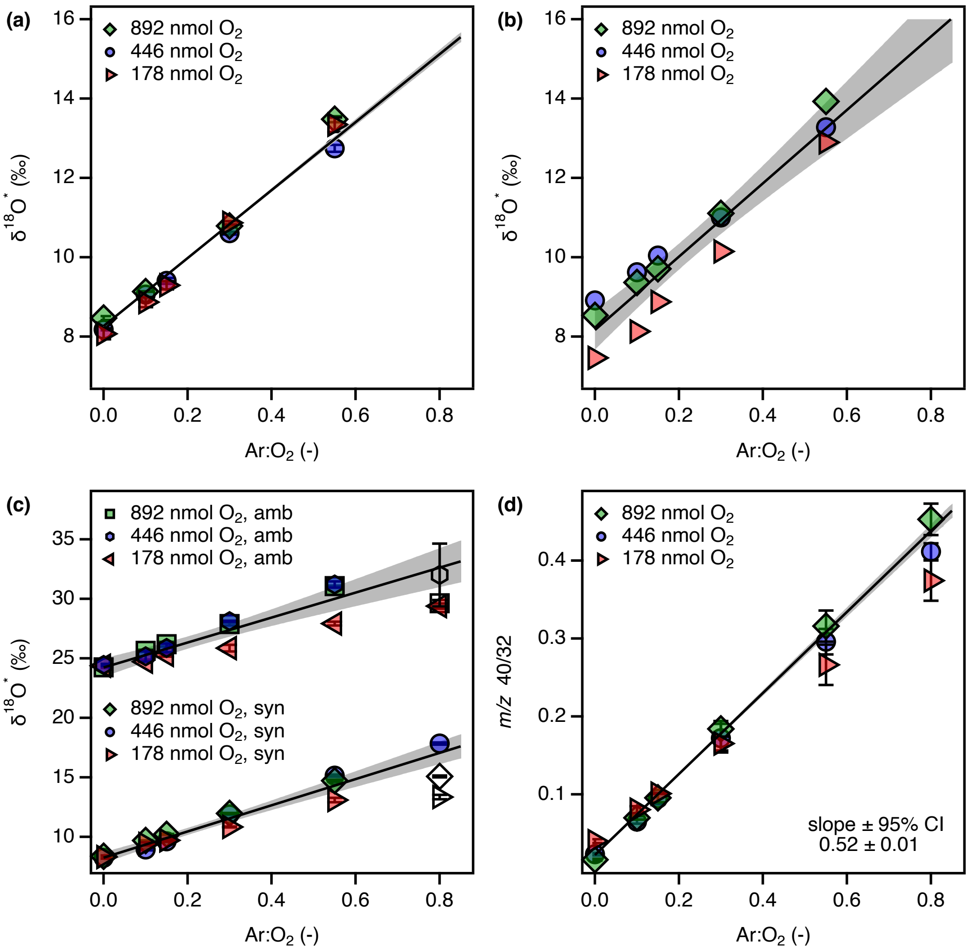

Figure S2 Effect of Ar interference shown as Ar:O_2 ratios on $^{18}O/^{16}O$ ratio measurements by GC/IRMS reported as $\delta^{18}O$ for gaseous samples of various O_2 concentrations. The instrument parameters for each panel are summarized in Table S2. Error bars correspond to standard deviations of triplicate measurements. Empty symbols were not included in the linear fit.

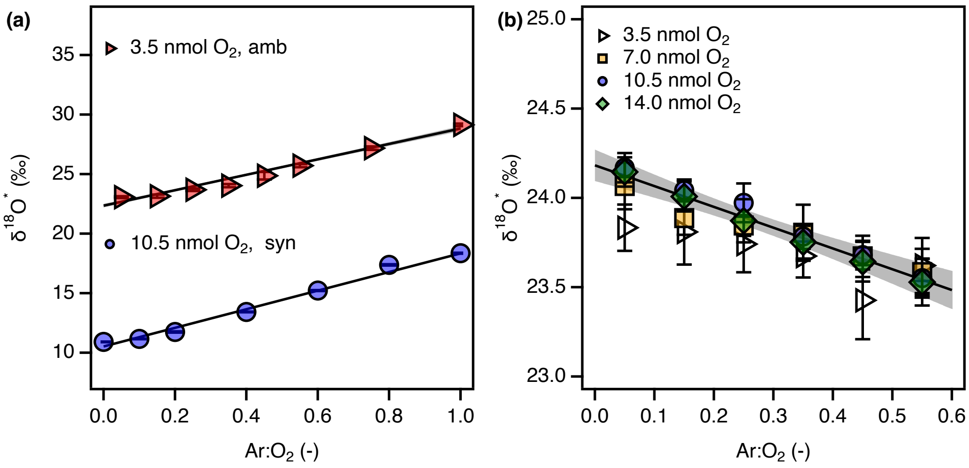

Figure S3 Effect of Ar interference shown as Ar:O_2 ratios on $^{18}O/^{16}O$ ratio measurements by GasBench/IRMS reported as $\delta^{18}O$ for gaseous samples of various O_2 concentrations. The instrument parameters for each panel are summarized in Table S3. Error bars correspond to standard deviations of seven replicate measurements. Empty symbols were not included in the linear fit.

S4 Manual peak integration

Because automatic peak integration did not distinguish between the Ar and O_2 peaks, we performed manual peak integration. Figure S4 shows an example of a typical chromatogram of a gaseous sample with an Ar:O_2 ratio of 0.55 on a 30 m GC/IRMS setup. While automatic peak integration includes both peaks (red bar), manual integration started at the minimum between the two peaks (green bar).

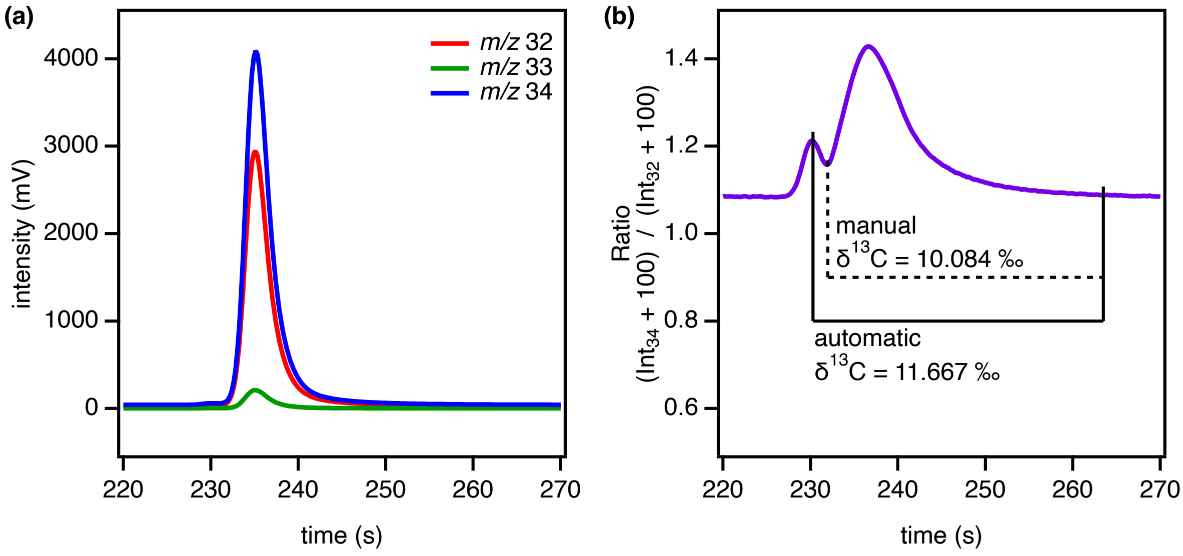

Figure S4 Chromatogram of a gaseous sample with an Ar:O_2 ratio of 0.55 in Isodat 3.0. The intensities of *m/z* 32, 33, and 34 are shown in panel (a) with the Ar peak eluting shortly before the main O_2 peak. The interference is more distinct in the mass ratio of 34/32 (b) with automatic peak integration times indicated by the solid black line and manual integration by the dashed black line.

S5 Quantification of Ar:O_2 ratios

The concentrations of Ar and O_2 and thus the Ar:O_2 ratio in the gaseous 3 mL headspace (subscript g) created in aqueous samples according to procedures described in Pati et al.[2] was calculated on the basis of equilibrium air-water partitioning.[7] The gaseous and aqueous concentrations of both Ar and O_2 in this two-phase system were determined by the initial aqueous concentrations of each species i, $c_{i,w}$, in the sample *before* creating a N_2 headspace. The gas phase concentrations of species i in the sample headspace (superscript $^{\boxminus}$), $c_{i,g}^{\boxminus}$, follow from the mass fraction in gaseous phase, f_g, which are determined by the gas-water volume ratios and the dimensionless Henry's law constant, $K_{i,\mathrm{H}}$, as in eq. S9. The mass fractions are multiplied by a volume ratio (v_w/v_g) to account for the fact that the total mass of O_2 in the sample vial (12 mL) originates from the aqueous phase sample only ($v_w = 9$ mL).

$$c_{i,g}^{\ominus} = c_{i,w} \cdot \frac{v_w}{v_g} \cdot f_{i,g} \tag{S9}$$
$$= c_{i,w} \cdot \frac{v_w}{v_g} \cdot \frac{c_{i,g}^{\ominus} v_g}{c_{i,g}^{\ominus} v_g + c_{i,w}^{\ominus} v_w}$$
$$= c_{i,w} \cdot \frac{v_w}{v_g} \cdot \frac{1}{1 + v_w/v_g \cdot 1/K_{i,\mathrm{H}}}$$

where v_w and v_g are the volumes of aqueous and gas phase in the sample vial. The Ar:O_2 ratio follows from the ratio of gas phase concentrations (eq. S10).

$$\frac{c_{\mathrm{Ar},g}^{\ominus}}{c_{\mathrm{O_2},g}^{\ominus}} = \frac{c_{\mathrm{Ar},w}}{c_{\mathrm{O_2},w}} \cdot \frac{f_{\mathrm{Ar},g}}{f_{\mathrm{O_2},g}} \tag{S10}$$
$$\mathcal{P} = \frac{c_{\mathrm{Ar},w} \cdot f_{\mathrm{Ar},g}}{f_{\mathrm{O_2},g}} \tag{S11}$$
$$= c_{\mathrm{Ar},w} \cdot \frac{1 + v_w/v_g \cdot 1/K_{\mathrm{O_2},\mathrm{H}}}{1 + v_w/v_g \cdot 1/K_{\mathrm{Ar},\mathrm{H}}}$$
$$\frac{c_{\mathrm{Ar},g}^{\ominus}}{c_{\mathrm{O_2},g}^{\ominus}} \equiv \frac{c_{\mathrm{Ar}}}{c_{\mathrm{O_2}}} = \frac{\mathcal{P}}{c_{\mathrm{O_2},w}} \tag{S12}$$

where $\mathcal{P}$ in eq. S11 is a constant defined by the aqueous to gaseous volume ratio (9 mL/3 mL), $K_{\mathrm{Ar,H}}$ (31.4, 20°C), and $K_{\mathrm{O_2,H}}$ (29.2, 20°C).[8] The Ar concentration in the aqueous phase before partitioning was assumed constant at 13.6 µM based on its partial pressure of 0.39 mol m^{-3}.[9] Note that for the sake of simplicity, subscript g and superscripts $^{\ominus}$ for the Ar:O_2 ratio in the headspace are omitted throughout the main manuscript and the SI for simplicity, as indicated in eq. S12.

S6 Consequences of Ar interferences on oxygen isotope enrichment factors of O_2

S6.1 Derivation of theoretical relationship

Changes in $\delta^{18}O$ of O_2 due to O_2 reduction follow from the general Rayleigh equation (eq. 3 from the main manuscript). The $\delta^{18}O$ of O_2 in an aqueous sample is determined by its initial O isotope signature, $\delta^{18}O_0$, the fraction of remaining dissolved O_2, $c_{\mathrm{O_2},w}/c^0_{\mathrm{O_2},w}$, and the enrichment factor, ε_{O}, of the O_2 reduction reaction according to eq. S13.

$$\delta^{18}\mathrm{O} = (\delta^{18}\mathrm{O}_0 + 1) \cdot \left(\frac{c_{\mathrm{O_2},w}}{c^0_{\mathrm{O_2},w}}\right)^{\varepsilon_{\mathrm{O}}} - 1 \tag{S13}$$

Without correction, the measured value, $\delta^{18}O^{\star}$, is increased by Ar interference according to the ratio of Ar to O_2, $c_{\mathrm{Ar}}/c_{\mathrm{O_2}}$, and the instrument specific correction factor, b, as defined in eq. 1

of the main manuscript.

$$\delta^{18}\mathrm{O}^{\star} = (\delta^{18}\mathrm{O}_0 + 1) \cdot \left(\frac{c_{\mathrm{O}_2,w}}{c^0_{\mathrm{O}_2,w}}\right)^{\varepsilon_\mathrm{O}} - 1 + b \cdot \frac{c_{\mathrm{Ar}}}{c_{\mathrm{O}_2}} \tag{S14}$$

In order to express the Ar enrichment as a function of the fraction of remaining dissolved O_2, equation S12 is transformed to include the initial dissolved oxygen concentration in the t_0 sample, $c^0_{\mathrm{O}_2,w}$.

$$\frac{c_{\mathrm{Ar}}}{c_{\mathrm{O}_2}} = \frac{\mathcal{P}}{c_{\mathrm{O}_2,w}} = \frac{\mathcal{P}}{c^0_{\mathrm{O}_2,w} \cdot c_{\mathrm{O}_2,w}/c^0_{\mathrm{O}_2,w}} \tag{S15}$$

Thus, the measured $\delta^{18}O$ can be expressed as a function of ε_O and $c_{\mathrm{O}_2,w}/c^0_{\mathrm{O}_2,w}$ (eq. 6 in the main manuscript).

$$\delta^{18}\mathrm{O}^{\star} = (\delta^{18}\mathrm{O}_0 + 1) \cdot \left(\frac{c_{\mathrm{O}_2,w}}{c^0_{\mathrm{O}_2,w}}\right)^{\varepsilon_\mathrm{O}} - 1 + \frac{b \cdot \mathcal{P}}{c^0_{\mathrm{O}_2,w} \cdot c_{\mathrm{O}_2,w}/c^0_{\mathrm{O}_2,w}} \tag{S16}$$

When uncorrected $\delta^{18}O^{\star}$ values are used to derive the $\varepsilon_O{}^{\star}$ of the reaction, they introduce an error, $\Delta\varepsilon_O$, described by the difference to the "true" value of ε_O without Ar interferences.

$$\Delta\varepsilon_\mathrm{O} = \varepsilon_\mathrm{O} - \varepsilon^{\star}_\mathrm{O} \tag{S17}$$

$\varepsilon^{\star}_O$ strongly depends on the O_2 turnover and number of samples withdrawn because Ar interference increases with O_2 consumption and thus increasing Ar:O_2 ratio. Low O_2 turnover samples thus lead to smaller $\Delta\varepsilon_O$ to an extent that depends on their weighting in the evaluation method. For simplicity and as a worst case scenario, we assume an experiment in which only two samples (measurements) are used to derive $\varepsilon^{\star}_O$. The first sample corresponds to the initial $\delta^{18}O^{\star}$ prior to O_2 conversion, $\delta^{18}O^{\star}_0$. The second sample is taken at the end of the reaction and designated $\delta^{18}O^{\star}_{max}$. Here, we determine $\varepsilon^{\star}_O$ from a linear form of eq. 3 of the main manuscript based on $\delta^{18}O^{\star}_0$ and $\delta^{18}O^{\star}_{max}$ as in eq. S18.

$$\varepsilon_\mathrm{O}{}^{\star} = \frac{\ln(\delta^{18}\mathrm{O}^{\star}_{\mathrm{max}} + 1) - \ln(\delta^{18}\mathrm{O}^{\star}_0 + 1)}{\ln(c_{\mathrm{O}_2,w}/c^0_{\mathrm{O}_2,w})_{\mathrm{max}} - \ln(c_{\mathrm{O}_2,w}/c^0_{\mathrm{O}_2,w})_0} \tag{S18}$$

$$= \frac{\ln(\delta^{18}\mathrm{O}^{\star}_{\mathrm{max}} + 1) - \ln(\delta^{18}\mathrm{O}^{\star}_0 + 1)}{\ln(c_{\mathrm{O}_2,w}/c^0_{\mathrm{O}_2,w})_{\mathrm{max}}} \tag{S19}$$

$$\Delta\varepsilon_\mathrm{O} = \varepsilon_\mathrm{O} - \frac{\ln(\delta^{18}\mathrm{O}^{\star}_{\mathrm{max}} + 1) - \ln(\delta^{18}\mathrm{O}^{\star}_0 + 1)}{\ln(c_{\mathrm{O}_2,w}/c^0_{\mathrm{O}_2,w})_{\mathrm{max}}} \tag{S20}$$

Insertion of eq. S16 for $\delta^{18}O^{\star}$ into eq. S20 leads to eq. S21 for $\Delta\varepsilon_O$. $\Delta\varepsilon_O$ now depends on the two variables ε_O and $c_{\mathrm{O}_2,w}/c^0_{\mathrm{O}_2,w}$. Note that both the initial and the final $\delta^{18}O$ measured is affected by Ar interference.

$$\Delta\varepsilon_O = \varepsilon_O - \frac{\ln\Big((\delta^{18}O_0 + 1)\cdot(c_{O_2,w}/c^0_{O_2,w})^{\varepsilon_O}_{\max} + (b\cdot\mathcal{P})/(c^0_{O_2,w}\cdot(c_{O_2,w}/c^0_{O_2,w})_{\max})\Big)}{\ln(c_{O_2,w}/c^0_{O_2,w})_{\max}}$$

$$- \frac{\ln\Big((\delta^{18}O_0 + 1) + (b\cdot\mathcal{P})/(c^0_{O_2,w})\Big)}{\ln(c_{O_2,w}/c^0_{O_2,w})_{\max}} \tag{S21}$$

S6.2 Illustrative calculations

We illustrate the consequence of Ar interferences onto the quantification of O isotope fractionation in O_2 reduction experiments with different ε_O. To that end, we derived the magnitude of $\varepsilon^\star_O$ in theoretical experiments using eq. S14 to calculate $\delta^{18}O^\star$. We assumed a typical experiment to consist of 10 separate O_2 samples withdrawn at different extent of O_2 conversion and a maximum O_2 conversion of 90%.

We calculated $\delta^{18}O^\star$ assuming three different ε_O-values, namely 0‰, −15‰, and +15‰ shown in Figure panels S5a, S5b, and S5c, respectively. These $\delta^{18}O^\star$ data points were subsequently used to derive $\varepsilon^\star_O$ and their 95% confidence intervals using non-linear regression with eq. 3 of the main manuscript. Figure S5 also shows $\delta^{18}O$ calculated with these ε_O-values in the absence of Ar interferences. The illustrative calculations show that $\Delta\varepsilon_O$ amounts to 1.3‰ in all cases. In case of no or normal O isotope fractionation, $\varepsilon^\star_O$ overestimate ε_O. In case of inverse O isotope fractionation, the effect is reversed and $\varepsilon^\star_O$ underestimates ε_O. Note that a reversion of the inverse O isotope fractionation trend for ε_O = 15‰ is only reversed at O_2 turnover exceeding 99%.

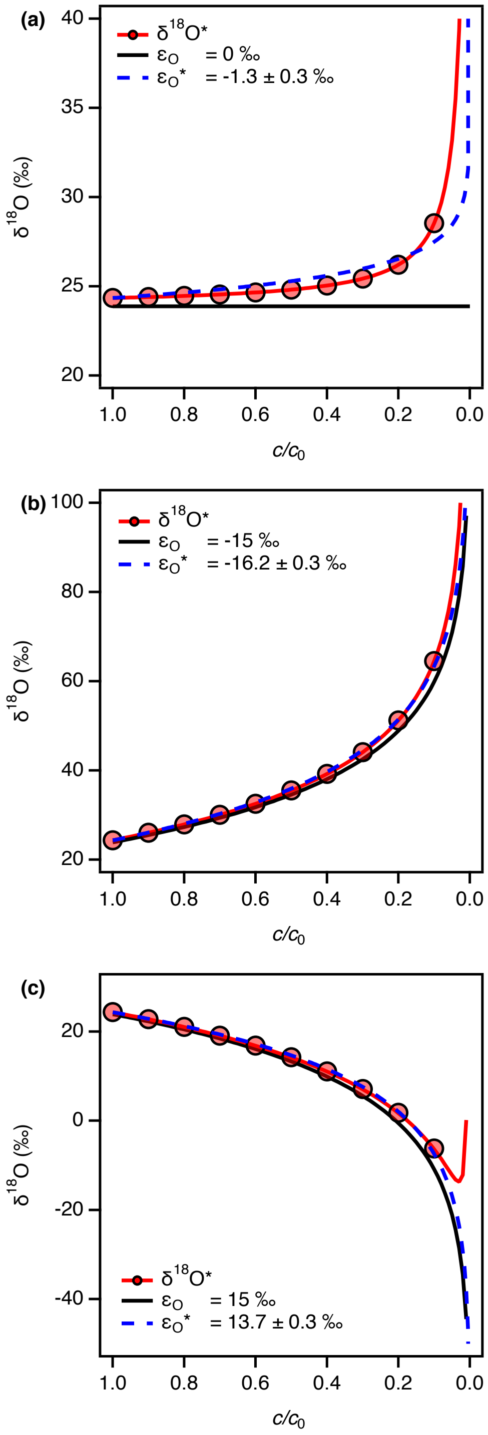

Figure S5 Illustrative calculations of $\delta^{18}O^{\star}$ and $\delta^{18}O$ in O isotope fractionation experiments where O_2 reduction is carried out with arbitrary "true" ε_O-values of $0‰$ (a), $-15‰$ (b), and $+15‰$ (c), respectively. $\varepsilon_O^{\star}$ were calculated from non-linear regression of the 10 data points for $\delta^{18}O^{\star}$ with eq. 3 of the main manuscript with a fixed initial $\delta^{18}O$.

S7 O_2 reduction by glucose oxidase

Figure S6 shows the increasing depletion of O_2 by glucose oxidase with increasing amounts of glucose. Initial concentrations of 270 and 255 μM of O_2 decreased by 0.81 and 0.69 μM per μM of glucose in buffer saturated with ambient and synthetic air, respectively. Consequently, Ar:O_2 ratios increased from 0.05 to 0.15 in the Ar-saturated samples based on eq. S12.

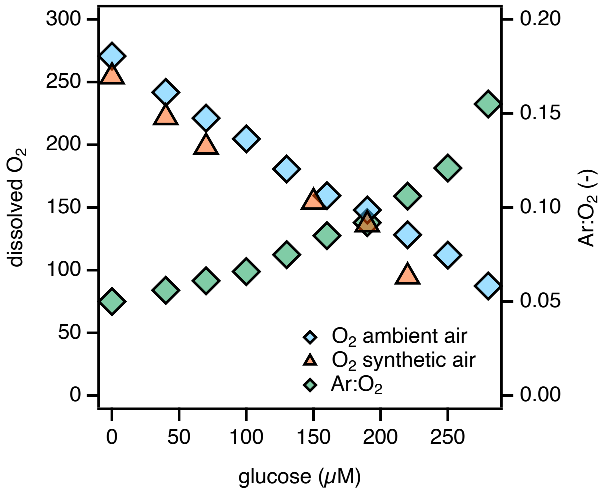

Figure S6 O_2 depletion in glucose oxidation experiments in buffer saturated with ambient and synthetic air. As a consequence, Ar:O_2 ratios increase.

S8 Estimation of Argon interference in measurements with lower detection limits

To illustrate the relevance of maximum turnover observed in a real O_2 consumption experiment in relation to the MDL, we reassessed data from Pati et al.[1]. The authors determined ε_O of O_2 reduction by glucose oxidase and Fe^{2+} based on single injection measurements of $\delta^{18}O$ on a GC/IRMS without accounting for Ar interference. Single injections lowered the MDL to 86 μM O_2 in the headspace or 16 μM dissolved O_2 in aqueous samples. Based on initial dissolved O_2 concentrations of about 230 μM, the theoretical maximum turnover within the MDL of 16 μM would have been 93% with an Ar:O_2 of 0.848 (eq. S12). In reality, the maximum turnover was about 80% for both experiments resulting in an Ar:O_2 of about 0.295 (eq. S12). While we cannot retrospectively determine the exact correction factor, b, of Ar interference on $\delta^{18}O^\star$, it is reasonable to assume a value comparable to the one we assessed for the same instrument setup (b of 8.57 ± 0.16, Table S2). Based on equation S14, we would expect deviations of $\delta^{18}O^\star$ from the real value in the maximum turnover sample of 2.5 and 7.3 ‰ for the real sample (c/c_0 = 0.2) and the sample at detection limit ($c/c_0 = 0.07$), respectively.

Each O_2 consumption experiment consisted of seven samples at different fractions of conversion which reduces the extent of Ar interference in the determination of ε_O. As discussed

in the main manuscript, the error is most distinct in reactions with a small ε_O. The ε_O of O_2 reduction by Fe^{2+} determined by Pati et al.[1] was -15.0 ± 0.17‰ which was significantly higher than previously reported values between 7.3 and 10.3‰.[10] While both values were analyzed on an CF-IRMS disregarding Ar interference, we previously saw that different setups can have very different extents of Ar interference that might account for the difference in ε_O (Table S1). Based on equation S21, however, the maximum error, $\Delta\varepsilon_O$, is 2.1 ‰, even for a maximum conversion of 93% and just 1.1 ‰ for the real sample at 80% conversion. Therefore, Ar interference is not responsible for the discrepancy of observed ε_O. For glucose oxidation, Pati et al.[1] determined an ε_O of 35.5 ± 3.7 ‰ which is significantly lower than the result of this study both with (-43.9 ± 3.4) and without Ar inerference (-43.2 ± 3.5, Table 1 of the main manuscript). Again, the maximum $\Delta\varepsilon_O$ is 2.0 and 1.0‰ for samples at 93 and 80% conversion respectively. Both examples manifest the conclusion that errors introduced by Ar interference are minor compared to other sources of error but should nevertheless be corrected for whenever feasible.

S9 References

[1] Pati, S. G.; Bolotin, J.; Brennwald, M. S.; Kohler, H.-P. E.; Werner, R. A.; Hofstetter, T. B. Measurement of oxygen isotope ratios ($^{18}O/^{16}O$) of aqueous O_2 in small samples by gas chromatography/isotope ratio mass spectrometry. *Rapid Commun. Mass Spectrom.* **2016**, *30*, 684–690, https://doi.org/10.1002/rcm.7481.

[2] Pati, S. G.; Kohler, H.-P. E.; Hofstetter, T. B. In *Measurement and Analysis of Kinetic Isotope Effects*; Harris, M. E., Anderson, V. E., Eds.; Academic Press, 2017; pp 292–329, https://doi.org/10.1016/bs.mie.2017.06.044.

[3] Barkan, E.; Luz, B. High-precision measurements of $^{17}O/^{16}O$ and $^{18}O/^{16}O$ of O_2 and O_2/Ar ratio in air. *Rapid Commun. Mass Spectrom.* **2003**, *17*, 2809–2814, http://dx.doi.org/10.1002/rcm.1267.

[4] Abe, O.; Yoshida, N. Partial pressure dependency of $^{17}O/^{16}O$ and $^{18}O/^{16}O$ of molecular oxygen in the mass spectrometer. *Rapid Commun. Mass Spectrom.* **2003**, *17*, 395–400, https://doi.org/10.1002/rcm.923.

[5] Sarma, V. V. S. S.; Abe, O.; Saino, T. Chromatographic Separation of nitrogen, argon, and oxygen in dissolved air for determination of triple oxygen isotopes by dual-inlet mass spectrometry. *Anal. Chem.* **2003**, *75*, 4913–4917, https://doi.org/10.1021/ac034314r.

[6] Jurikova, H.; Guha, T.; Abe, O.; Shiah, F.-K.; Wang, C.-H.; Liang, M.-C. Variations in triple isotope composition of dissolved oxygen and primary production in a subtropical reservoir. *Biogeosciences* **2016**, *13*, 6683–6698, http://dx.doi.org/10.5194/bg-13-6683-2016.

[7] Schwarzenbach, R. P.; Gschwend, P. M.; Imboden, D. M. *Environmental Organic Chemistry*, 3rd ed.; John Wiley & Sons, 2017; p 1005.

[8] Sander, R. Compilation of Henry's law constants (version 4.0) for water as solvent. *Atmos. Chem. Phys.* **2015**, *15*, 4399–4981, https://doi.org/10.5194/acp-15-4399-2015.

[9] Berner, E. K.; Berner, R. A. *Global environment: water, air, and geochemical cycles*; Princeton University Press, 2012.

[10] Oba, Y.; Poulson, S. R. Oxygen isotope fractionation of dissolved oxygen during reduction by ferrous iron. *Geochim. Cosmochim. Acta* **2009**, *73*, 13–24, http://dx.doi.org/10.1016/j.gca.2008.10.012.
